# Structure-Based Alignment and Consensus Secondary Structures for Three HIV-Related RNA Genomes

**DOI:** 10.1371/journal.pcbi.1004230

**Published:** 2015-05-20

**Authors:** Christopher A. Lavender, Robert J. Gorelick, Kevin M. Weeks

**Affiliations:** 1 Department of Chemistry, University of North Carolina, Chapel Hill, Chapel Hill, North Carolina, United States of America; 2 AIDS and Cancer Virus Program, Leidos Biomedical Research, Inc., Frederick National Laboratory for Cancer Research, Frederick, Maryland, United States of America; University of Texas at Austin, UNITED STATES

## Abstract

HIV and related primate lentiviruses possess single-stranded RNA genomes. Multiple regions of these genomes participate in critical steps in the viral replication cycle, and the functions of many RNA elements are dependent on the formation of defined structures. The structures of these elements are still not fully understood, and additional functional elements likely exist that have not been identified. In this work, we compared three full-length HIV-related viral genomes: HIV-1_NL4-3_, SIVcpz, and SIVmac (the latter two strains are progenitors for all HIV-1 and HIV-2 strains, respectively). Model-free RNA structure comparisons were performed using whole-genome structure information experimentally derived from nucleotide-resolution SHAPE reactivities. Consensus secondary structures were constructed for strongly correlated regions by taking into account both SHAPE probing structural data and nucleotide covariation information from structure-based alignments. In these consensus models, all known functional RNA elements were recapitulated with high accuracy. In addition, we identified multiple previously unannotated structural elements in the HIV-1 genome likely to function in translation, splicing and other replication cycle processes; these are compelling targets for future functional analyses. The structure-informed alignment strategy developed here will be broadly useful for efficient RNA motif discovery.

## Author Summary

Human immunodeficiency virus (HIV) is a persistent and critical threat to human health. Replication and pathogenesis of HIV is governed by information encoded in its single-stranded RNA genome. In addition to coding for viral proteins, the HIV genomic RNA forms base paired and higher-order structures that are critical for viral replication. It is likely that only a subset of functional RNA motifs has been identified. Here, we interrogate the structures of three diverse HIV-related viral genomes by nucleotide-resolution chemical probing. The three genomes include HIV-1, the virus that infects humans, and SIVcpz and SIVmac, which are progenitors for the main branches of the two HIV evolutionary groups. We used a structure-informed alignment approach to generate consensus models for base-paired secondary structures that are shared by these three HIV-related genomes. With this approach, we were able to recapitulate all known RNA structures and, additionally, discovered multiple previously undescribed structural elements that are clearly conserved among major HIV groups. We anticipate that the methods described here will be broadly useful for RNA structure motif discovery and, more immediately, for identification of RNA targets in HIV that are promising sites for therapeutic intervention.

## Introduction

RNA plays a direct role in most biological processes [1], and multiple examples of RNA function are found in the replication cycles of positive-strand RNA lentiviruses [2]. Viral RNA genomes function at two distinct levels: in the linear encoding of protein sequences and in functional higher-order RNA structures. Constrained by a small genome size, these viruses make efficient use of limited genome space in terms of both sequence allocation and densely arranged regulatory RNA structures.

RNA elements in the human immunodeficiency virus (HIV) genome play important regulatory roles throughout the replication cycle. During transcription of the integrated viral genome, a stem-loop structure in the 5ʹ untranslated region (UTR), called TAR, binds the Tat protein to recruit proteins involved in transcription [3, 4]. In the *env* gene, the Rev response element (RRE) binds the viral Rev protein, allowing unspliced and partially spliced viral mRNA to be exported out of the nucleus [5]. During translation, the *gag-pol* frameshift element modulates the reading frame of the ribosome, tightly regulating production of the Gag-Pol polypeptide [6, 7]. Stem-loop structures in the Psi packaging element are required for efficient packaging of viral genome into nascent virions [8]. Multiple pseudoknots modulate replicative functions [9]. Although most structural characterization of HIV-related RNA genomes has focused on the 5ʹ and 3ʹ untranslated regions, recent analyses make clear that the central coding region of HIV genomes has extensive potential to base pair and form higher-order RNA structures [10, 11]. Although the importance of many structured RNA elements is supported by direct experimental validation, the functional significance of many other RNA structures is unknown. More broadly, it remains difficult to rigorously identify conserved RNA structure motifs when sequence conservation is low without meticulous hand-alignment of annotated sequences.

SHAPE chemical probing makes possible powerful and direct experimental interrogation of higher-order RNA structure. In the SHAPE approach, a structurally selective electrophile is used to acylate the 2ʹ-hydroxyl of unstructured or conformationally dynamic RNA nucleotides [12]. The extent of modification is roughly inversely proportional to the tendency of an RNA nucleotide to participate in an RNA base pair or other structural interaction. SHAPE has recently been adapted to readout by massively parallel sequencing using mutation profiling. Mutational profiling, or MaP, exploits the ability of reverse transcriptase to extend through the site of a chemical lesion in RNA and to record the RNA modification as a sequence change in the synthesized cDNA [9]. Chemical adduct-induced sequence changes can then be related to SHAPE reactivities on an absolute scale. The combined approach, called SHAPE-MaP, allows facile SHAPE-based structural characterization of complex RNA molecules and has thus far been applied to the RNA genomes of both HIV-1 [9] and hepatitis C virus [13].

**Fig 1:**
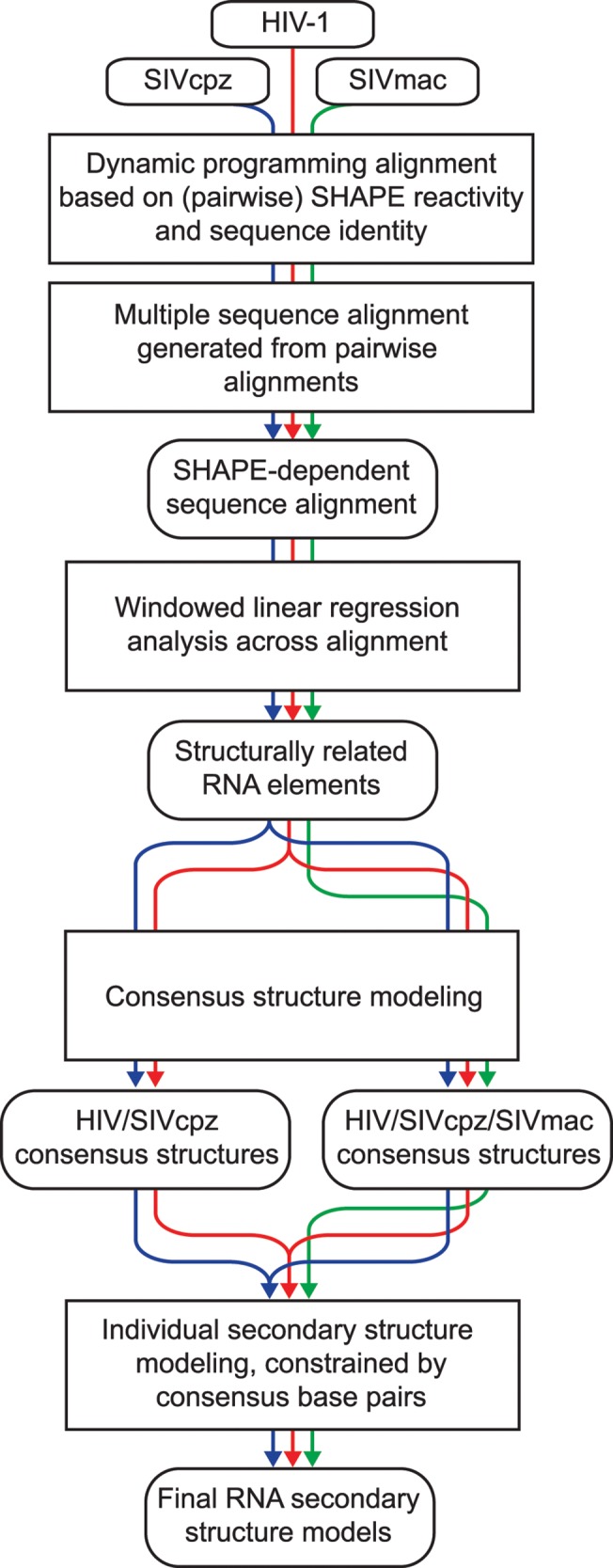
RNA structure-based comparative genome analysis. Sequence alignments were created through SHAPE-dependent pairwise comparisons, which were then combined into multiple sequence alignments [24]. Windowed linear regression analysis of SHAPE data was then used to define regions where structural conservation is implied by correlation of SHAPE reactivities. For these regions, consensus secondary structures were modeled using SHAPE- and sequence-dependent folding [50]. Consensus secondary structures were found for both HIV-1/SIVcpz and three-genome alignments. Base pairs that did not disagree between the two consensuses and that had a pairing probability greater than 95% were used to constrain a final model for HIV-1 using SHAPE-directed folding [29].

To address the functional significance of RNA structures in HIV-related genomes, we characterized the conservation of structural features across the genomes of HIV-1 (strain NL4-3) [14] and two related primate lentiviruses, SIVcpz MB897 [15] and SIVmac239 [16, 17]. Fig 1 illustrates the analysis workflow. Using comprehensive SHAPE-MaP chemical probing data from each of the three RNA genomes, structure-dependent sequence alignments were generated. We then identified areas in which chemical modification patterns were statistically correlated. Finally, we generated secondary structure models taking into account both SHAPE reactivities and sequence covariation. This analysis identified multiple regions of structural similarity across the three HIV-related strains that included all previously identified well-characterized RNA elements. Strikingly, we also identified multiple previously undescribed structural elements that are clearly conserved among HIV-1 and related viruses. These elements are compelling sites for follow-up functional studies and are potential therapeutic targets. Analysis is fully automated, and we anticipate that our structure-based sequence comparison strategy will see broad application as whole-transcriptome chemical probing data become available.

## Results

### Selection of virus strains

Viral strains were selected based on epidemiological importance and with respect to their divergence from reference strain NL4-3, a member of HIV-1 group M. SIVcpz MB897 (SIVcpz) infects chimpanzees, and the SIVcpz virus is thought to have given rise to the HIV-1 strains responsible for the worldwide AIDS epidemic [18, 19]. SIVmac239 (SIVmac) is derived from a virus that infects sooty mangabeys and is capable of infecting macaques. This strain is widely used as the reference strain for the SIVsm/HIV-2 lineage. Of the two strains studied, SIVmac is the more distantly related to HIV-1 group M strains [20]. SIVcpz and SIVmac have sequence identities of 77.4% and 54.6%, respectively, when compared to NL4-3 using standard sequence-based alignments.

### Genomic SHAPE-MaP

Whole-genome SHAPE data for HIV-1 were obtained previously [9], and SHAPE data for SIVcpz and SIVmac were generated for this work. Authentic SIVcpz and SIVmac genomic RNAs were purified from mature virions using a non-denaturing approach [21]. To preserve secondary and tertiary structures in the RNAs, no heating steps or chaotropic agents were used during RNA genome purification.

Chemical modification of the viral RNAs with SHAPE reagent 1-methyl-7-nitroisatoic anhydride (1M7) was performed under physiological-like ion conditions [12, 22]. Following chemical probing, the extent of SHAPE-adduct formation at each nucleotide was determined by massively parallel sequencing using mutational profiling [9]. SHAPE reactivity values were determined at each position by comparing mutation rates of a 1M7-modified sample relative to background controls. SHAPE reactivity is correlated with the flexibility of a given nucleotide; nucleotides with low SHAPE reactivity tend to participate in base pairs or other interactions, whereas nucleotides with high SHAPE reactivity tend to be in unstructured regions of the RNA. SHAPE reactivity measurements were made for an average of 98% of the nucleotides in each RNA genome (Supporting Information).

### SHAPE-dependent whole-genome alignments

**Fig 2:**
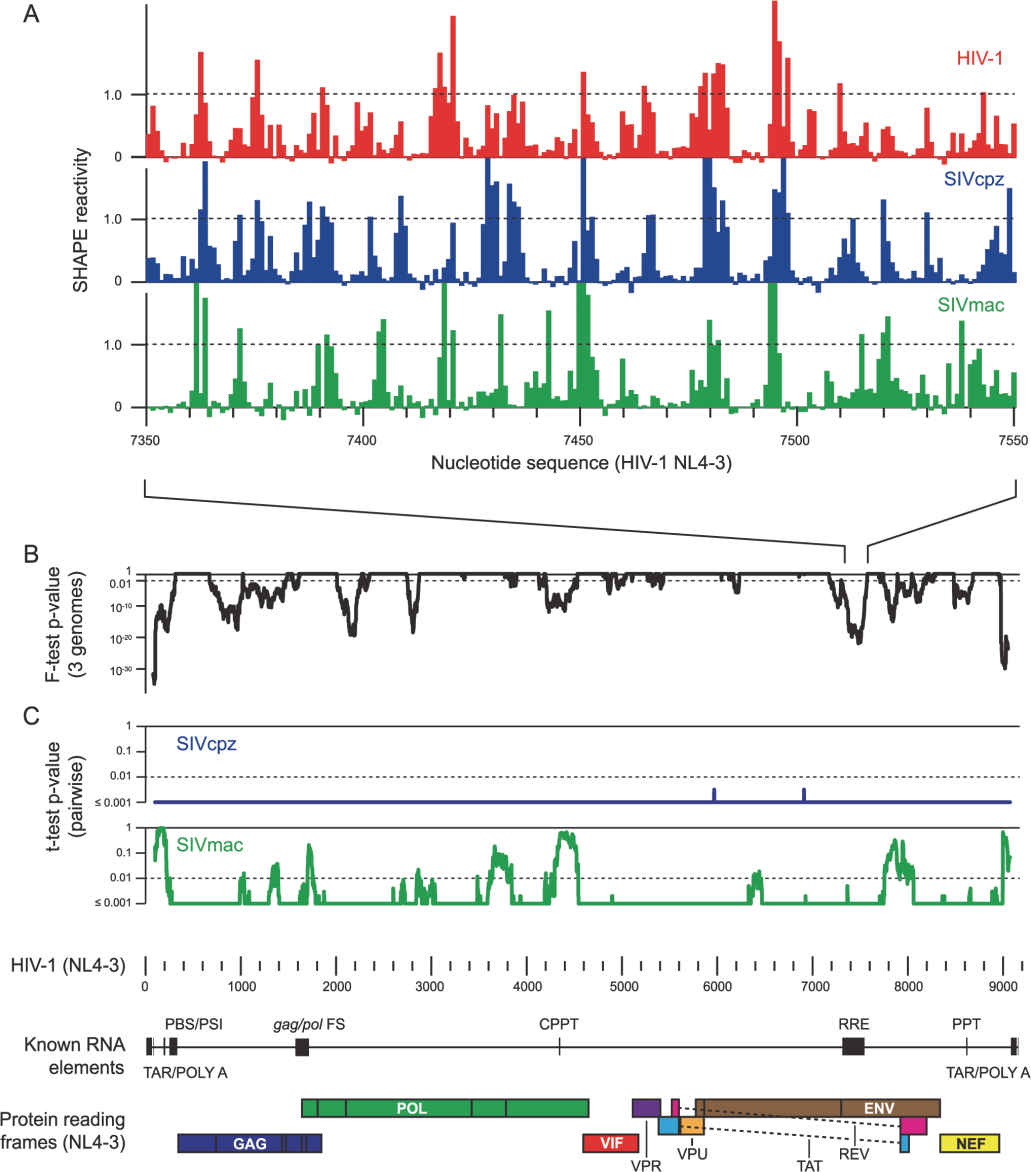
SHAPE-structure dependent alignment. (**A**) SHAPE-directed alignment over one 200-nt window in the RRE. Sequences are numbered relative to the HIV-1 RNA genome, with the transcription start site as +1. (**B**, **C**) Windowed linear regression statistics as a function of the HIV-1 (NL4-3) sequence, computed over 200-nt windows. Correlations of SHAPE values across the three-genome sequence alignment were evaluated by F-test (results shown as *p*-values; black); pairwise comparisons with HIV-1 were evaluated by t-test (results shown as *p*-values). The entire HIV-1 alignment is shown; RNA landmarks are given at the bottom of the figure.

Pairwise whole-genome alignments of HIV-1, SIVcpz, and SIVmac RNAs were determined by a SHAPE-dependent dynamic programming algorithm [23] (see preceding companion article in this issue). From these structurally-directed pairwise alignments, we generated a single, multiple-sequence alignment [24] (Fig 2A). All regions previously shown to contain functional RNA structures were aligned correctly by this fully automated approach. Aligned elements fell in both untranslated regions (for example, 5' and 3' TAR stems and the Psi packaging element) and coding regions (*gag-pol* frameshift element and the RRE). In addition, the polypurine tracts in the *pol* and *nef* genes (cPPT and PPT, respectively) [25, 26] aligned precisely.

The fully automated pairwise HIV-1 and SIVcpz alignment is highly accurate relative to the manually edited alignments in the Los Alamos National Laboratory (LANL) HIV database [27]. Despite not explicitly considering codon alignment, our pairwise SHAPE-structure based alignments have sum-of-pairs and column scores of 95.6% and 94.5%, respectively, relative to LANL alignments (sum-of-pairs and column scores report similarity to a reference alignment considering aligned position pairs and aligned columns, respectively). Relative to LANL alignments, the three-sequence SHAPE-dependent alignment considering HIV-1, SIVcpz, and SIVmac show sum-of-pairs and column scores of 75.5% and 56.9%, respectively. In general, areas of disagreement between the SHAPE-structure and the LANL alignments, based on windowed column scores, lie in regions of the HIV-1 sequence with multiple overlapping reading frames and in regions encoding the variable loops in the *env* gene (S1 Fig). It is not clear which alignments are actually superior in these regions. In addition, RNA structure and codon alignments may not be strongly conserved in these areas, due to the selective pressure of multiple reading frames or to high mutation rates in the variable sequence regions.

### Evaluation of correlation by multi-variable linear regression

To evaluate the relationships among SHAPE data for the three RNA genomes, multi-variable linear regression was performed across the three-genome multiple sequence alignment over 200-nucleotide windows (Fig 2). By identifying areas with correlated SHAPE reactivities (Fig 2A), we sought to find areas in the sequence alignment with conserved RNA structures. SHAPE data were fit to a three-dimensional linear regression model, and the correlations among the three HIV-related strains were evaluated using the F-test. Based upon F-statistic measurements, *p*-values defining the significance of the correlation were determined for SHAPE values over the entire alignment (see Methods). These *p*-values were used to identify structural elements conserved among all three RNA genomes (Fig 2B). In addition, a t-test was used to gauge the pairwise correlations between HIV-1 and SIVcpz or SIVmac (Fig 2C).

Multiple regions across the three-genome alignments showed significant interdependences in SHAPE reactivities (Fig 2B; defined as F-test *p*-value ≤ 0.01). Regions with statistically significant SHAPE reactivity correlations occurred both at the 5ʹ and 3ʹ ends and in internal coding regions. Without exception, all previously identified functional elements are located in regions with correlated SHAPE reactivities. Critically, there are also multiple regions of similarity where no functional RNA structures had previously been identified. These newly identified regions lie in the *gag*, *pol*, and *env* genes.

Generally, regions that are statistically significant by both pairwise t-test analyses coincided with regions that correlated across all three genomes by F-test (Fig 2C). As expected, the pairwise t-test analyses showed that statistically significant SHAPE reactivity correlations are more widespread for the HIV-1 and SIVcpz comparison than for the more distantly related HIV-1 and SIVmac viruses. There are defined regions where correlations by F-test across the three-genome alignment are not recapitulated in HIV-1 and SIVmac pairwise t-test analyses, most conspicuously near NL4-3 residues 4400 and 7900. These are likely areas of structural conservation between HIV-1 and SIVcpz that are not shared between HIV-1 and SIVmac.

### Consensus secondary structures

**Fig 3:**
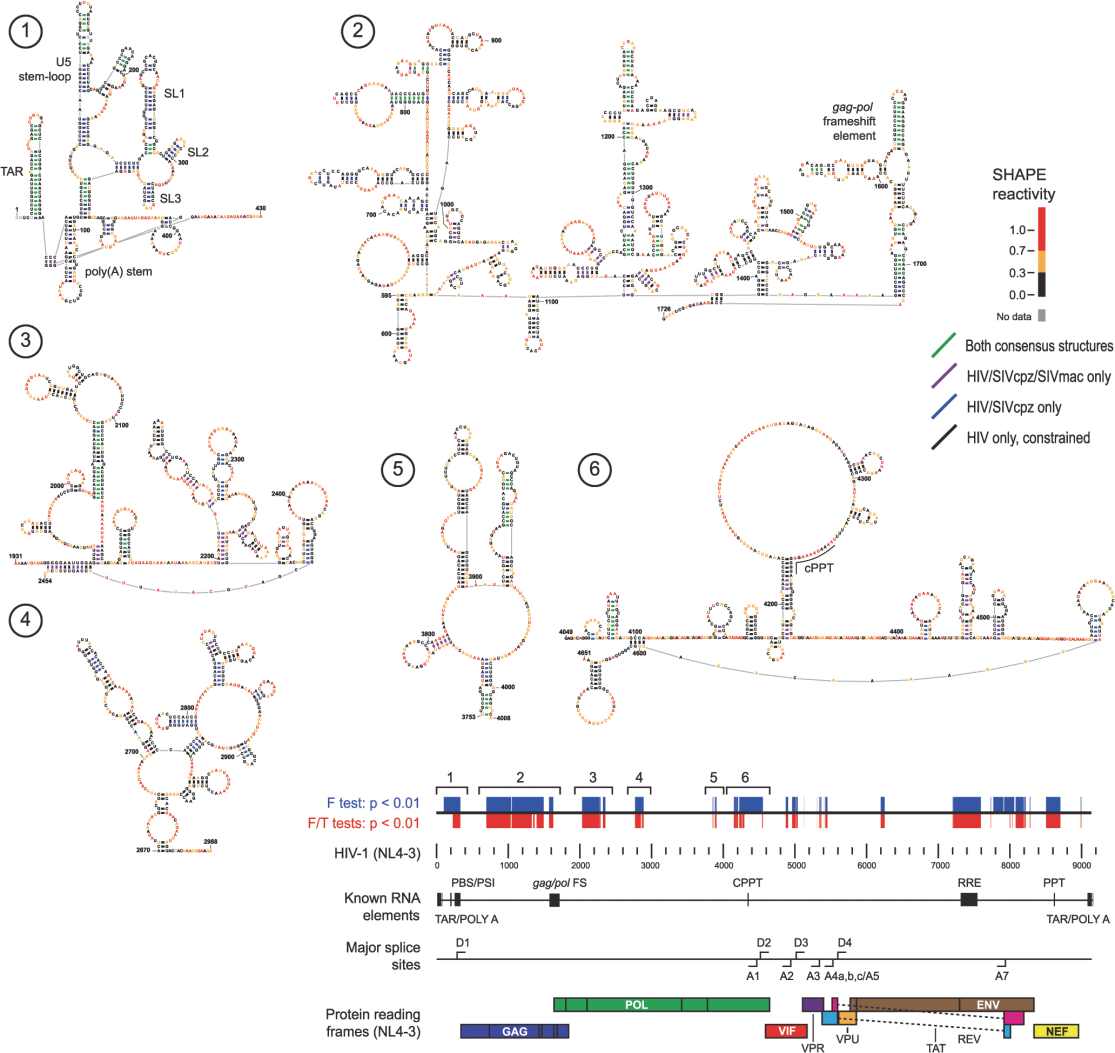
Secondary structure models for six structurally conserved elements in the 5' half of HIV-related RNA genomes. Nucleotides are colored by HIV-1 SHAPE reactivities. Secondary structures are shown for the final constrained HIV-1 secondary structure model. Base pairs are colored by level of structural consensus; black base pairs appear only in constrained HIV-1 predictions. Positions of predicted elements are shown on the HIV-1 (NL4-3) genome; annotations indicate statistical dependence, known RNA elements, major splice sites, and protein reading frames.

**Fig 4:**
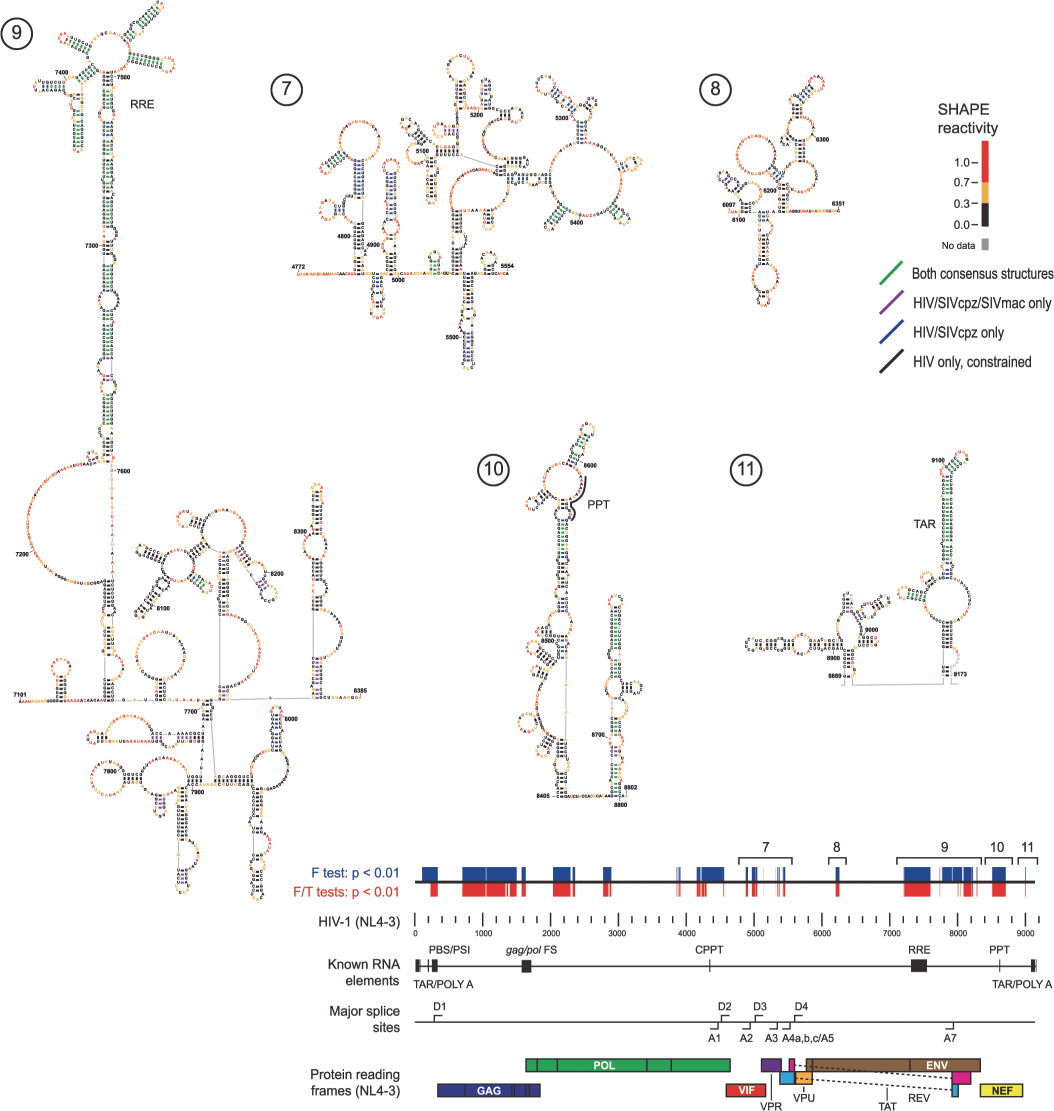
Secondary structure models for five structurally conserved elements in the 3' half of HIV-related RNA genomes. Secondary structures are shown for the final constrained HIV-1 secondary structure model. Other figure annotations are described in the Fig 3 legend.

Using the SHAPE-directed multiple sequence alignment, we developed consensus secondary structure models based on both nucleobase identity and SHAPE reactivity data [23] (Figs 3 and 4). Secondary structure models were generated for areas with statistically significant correlations (F-test *p*-value < 0.01). Consecutive regions with significant correlations were combined into single regions for structure modeling. Based on this criterion, eleven areas were selected from the three-genome alignment, ranging in length from 255 to 1285 nucleotides and collectively covering 68.4% of the HIV-1 NL4-3 genome.

**Fig 5:**
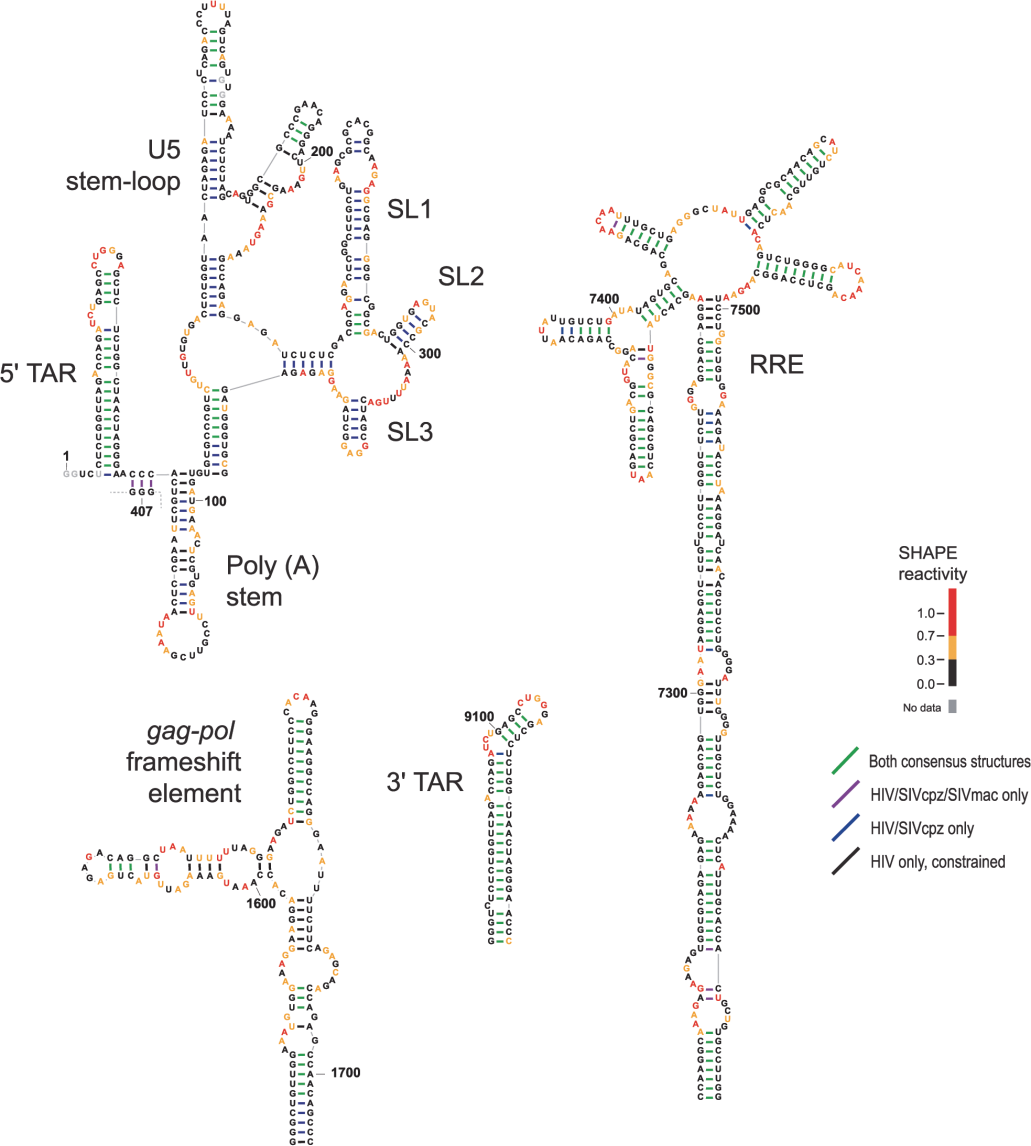
Secondary structure models for RNA elements with prior well-established functions. Secondary structures correspond to the final, fully automated constrained HIV-1 prediction. Other figure annotations are described in the Fig 3 legend.

Consensus secondary structures of regions with structural similarity implied by correlated SHAPE data were generated using three orthogonal inputs: RNA nearest-neighbor free energy rules, sequence covariation, and SHAPE reactivities [28–31]. For each region, two consensus structures were developed: one incorporating sequence alignment and SHAPE data for all three genomes and the other using pairwise information for only HIV-1 and SIVcpz, the two more closely related genomes. Consensus base pairs with pairing probabilities greater than 95% that did not disagree between the two consensus structures were then used to restrain a SHAPE-directed secondary structure model for HIV-1 [28, 32]. Consensus base pairs from the three-genome and HIV-1/SIVcpz comparisons are shown on the final constrained HIV-1 model (Figs 3 and 4). Consensus base pairs are highly over-represented in known functional elements (Fig 5). Importantly, consensus base pairs are also found in multiple areas with no previously identified function. In general, consensus base pairs occur more frequently at the 5ʹ and 3ʹ ends of the genome, though regions with notable consensus base pairing also occur in the central coding region.

## Discussion

### Secondary structure motifs conserved across diverse lentivirus lineages

Cellular and viral RNAs encode information in the form of higher-order RNA structures. The structures of a vast majority of transcribed RNAs are uncharacterized, and new strategies are needed to efficiently and rigorously search for functionally important structural elements. Here we applied an approach that makes possible motif discovery for elements whose function is implied by structural conservation; the approach is described in detail in the preceding companion manuscript [23]. First, model-free RNA structure comparisons were performed using whole-genome structure information experimentally derived from nucleotide-resolution SHAPE reactivities. Consensus secondary structures were then constructed for strongly correlated regions by taking into account both SHAPE probing structural data and nucleotide covariation information from structure-based alignments. We identified 314 base pairs with pairing probabilities greater than 95% that are shared between the two distinct consensus models developed in this work, one generated considering HIV-1, SIVcpz, and SIVmac RNA genomes and the other considering only the more closely related HIV-1 and SIVcpz sequences. Of these base pairs about half (171 base pairs, 54.5%) are in previously described elements. Strikingly, however, nearly as many base pairs with strong consensus support exist in regions with no known function (143 base pairs, 45.5%). That these structures are conversed across diverse HIV-related strains implies critical, if currently unknown, functionality.

The structural model developed in this work accurately reflects most of what is known about the genomic RNA structure of HIV-1 and related lentiviruses. Known functional elements are recapitulated precisely and are enriched with conserved base pairs (Fig 5). Our analysis provides additional perspective on these well-studied elements. For example, the TAR secondary structure for SIVmac forms two similar, but distinct, stem-loops [33]. The single stem-loop TAR elements from HIV-1 and SIVcpz align specifically with the second of the two stem-loops in SIVmac, suggesting greater structural similarity. Structural conservation as evidenced by correlated SHAPE reactivities is found throughout the HIV-1 genome including in the *env* and *pol* genes (Fig 2), consistent with studies implicating conserved RNA structure in these regions [34, 35]. A conserved structural element in the *pol* gene, at the protein domain junction between protease and reverse transcriptase, shares structural features with a previously identified structural element (Fig 3; nucleotides 2015–2121 in structure 3) [36].

This work also expands on the results of previous SHAPE-based analyses of HIV genomic RNAs. Recently, SHAPE-MaP studies focused on HIV-1 alone were used to model the whole-genome secondary structure [9]. Though that study and the work described here both ultimately resulted in secondary structure models, each approach has unique advantages. The prior work introduced the melded use of SHAPE reactivities and Shannon entropies to identify *de novo* regions with well-determined stable RNA structures based on information from a single sequence. This work uses evolutionary conserved sequence and structural alignment to identify regions with conserved structure and, additionally, to identify specific regions within larger motifs that are the most conserved structurally. The SIVmac genome has also been previously analyzed by SHAPE [11], and the resulting secondary structure model was compared to a SHAPE-directed secondary structure model for HIV-1 [10]. Model-based comparisons were made by hand, guided by manually edited sequence alignments. From this model-based manual comparison, 71 base pairs were identified as directly conserved between HIV-1 and SIVmac [11]. These base pairs are largely recapitulated in this work, including a stem-loop structure present at splice acceptor site one (called A1); perturbation of this structure dramatically impacts splicing at this site [11]. We do not see evidence of the shifting pairing partners in the RRE described in the prior SIVmac study. Despite not explicitly considering protein sequence, our structure-informed alignment preserves codon alignment at the RRE and accurately predicts its accepted secondary structure [11].

The strategy for structural motif discovery directed by fully automated SHAPE-based structure alignment created in this work is notably more successful than prior manual analyses, both at recapitulating known functional structures and in discovering new elements whose structures are conserved across diverse viral strains. This success is attributable to three features. First, the SHAPE-MaP approach itself is fully automated and avoids systematic errors or biases introduced by manual data processing required by prior capillary electrophoresis-based approaches. Second, sequence comparisons performed in this work directly consider a first-order metric of RNA structure—SHAPE reactivity—as opposed to base identity alone. Third, the approach created in this work performs these comparisons in a model-free way, avoiding complications arising from the complexity of large RNA secondary structure modeling.

Ultimately, the biological relevance of structure motifs identified in genome-wide studies must be examined and validated by direct experimentation. This work strongly constrains regions that merit such investigation. For example, in one recent study, four RNA hairpins were selected for mutational studies to determine their effects on viral replication [37]. Mutations in these four hairpins did not affect viral replication. Two of the four hairpins evaluated in the recent study (termed POL1 and POL3) fall in areas of insignificant structural correlation. A third (NEF1) has a different consensus-supported structure. The remaining hairpin (POL2) appears in the final HIV-1 structure model; however, this hairpin is not conserved in either the three- or two-genome consensus predictions. Thus, none of these hairpins would have been good candidates for mutagenesis studies and functional characterization based on the models developed in this work. In contrast and as described below, this work identifies multiple motifs that are compelling targets for future functional studies.

### Conserved, structured RNA elements at junctions between protein-coding domains

**Fig 6:**
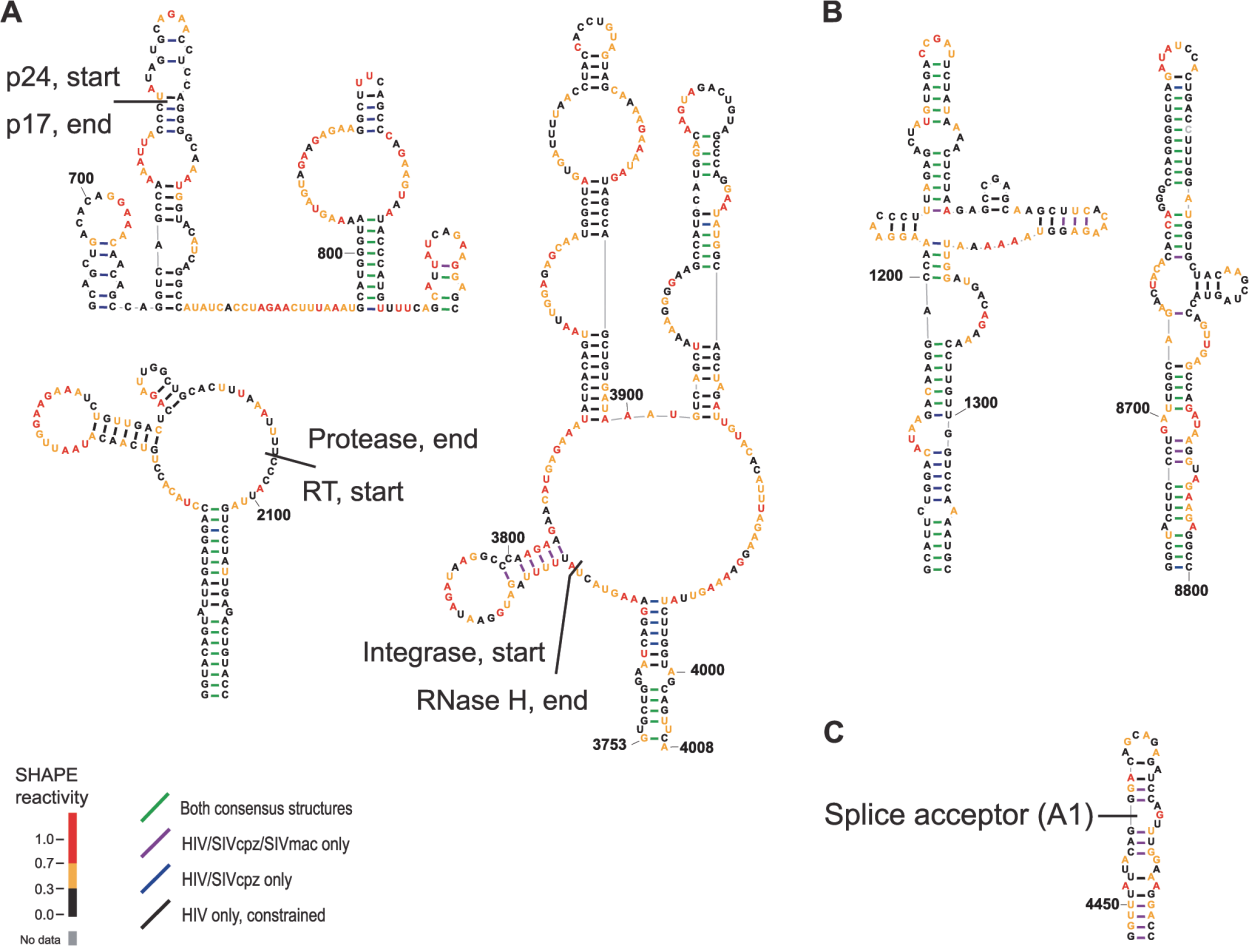
Novel conserved, likely functional, elements in the final HIV-1 structure model. (**A**) Structural elements located near protein-protein junctions. Protein domain junctions are labeled. (**B**) Conserved structural elements with the potential to form long (>20 bp) helical stacks. (**C**) Conserved structure at the A1 splice site, described in Pollom *et al*., recapitulated in this work [11].

Proteins fold co-translationally, and RNA structural stability affects ribosomal pausing during translation [38, 39]. Given that the extent of RNA structure formation influences pausing of the ribosome, local RNA structure could in turn modulate protein structure and associated activity [40]. Prior analysis of the HIV-1 genome revealed a potential relationship between highly structured regions of the RNA genome and the junctions between protein domains in HIV-1 polyprotein precursors [10]; however, it was not then possible to identify specific RNA structures that might define the relationship between RNA structure and translation. Here we observed that multiple conserved structured elements occur at or near protein-protein junctions and at protein inter-domain boundaries. Conserved structured elements occur in *gag* at the junction between p17 (matrix) and p24 (capsid) (Fig 6A, upper left), in *gag-pol* at the junction between protease and reverse transcriptase (Fig 6A, lower left), and between RNase H and integrase coding domains (Fig 6A, right). Base pairs in these regions show conservation in both the HIV-SIVcpz comparison and in the three-genome consensuses (Fig 6). In each proposed conserved structure element, conserved helices are present near the domain junction with a stable conserved helix occurring 3' of the protein domain junction.

### Two elements with extended conserved base pairing

Automated SHAPE-based alignment also identified structural elements that are clearly conserved among the three HIV-related strains but for which it is currently difficult to propose functions. Most strikingly, there are two regions that contain long helical elements with extensive conservation of base pairing (Fig 6B). Including potential stacking interactions, these elements contain helical elements extending for 20 or more base pairs.

### Structural features common to polypurine tract elements

Polypurine tracts are functionally critical retroviral sequence elements that act as RNA primers for positive-strand DNA synthesis [41]. How these elements function mechanistically is unknown. Following first-strand DNA synthesis, the PPT RNA-DNA hybrid duplex is preserved, and the rest of the RNA genome is degraded. Each of the retroviruses studied here has two distinct PPT sequences: the PPT sequence in the *nef* gene and the central polypurine tract (cPPT) in the *pol* gene.

**Fig 7:**
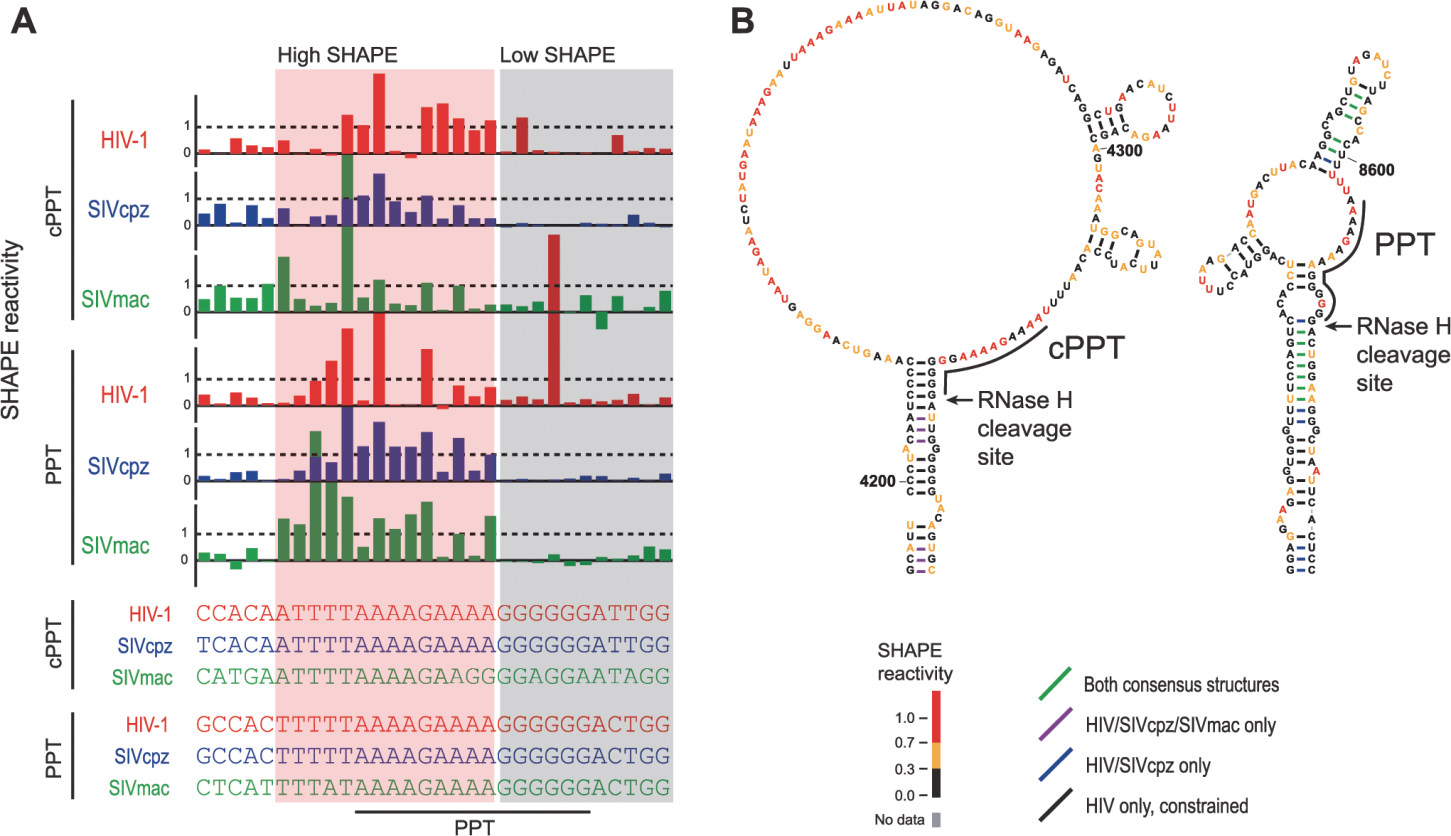
Consensus structures for the cPPT and PPT elements. (**A**) cPPT and PPT sequences and SHAPE reactivities. The six sequences correspond to two elements from each of three genomes. Regions with consistently low or high SHAPE reactivities are highlighted by shading. (**B**) Structure models for cPPT- and PPT-containing elements in the final HIV-1 model.

The cPPT and PPT regions were previously noted to have similar patterns of SHAPE reactivity based on analysis of SHAPE probing data for HIV-1 and SIVmac [11]. Consistent with this prior finding, we found that the polypurine sequences have a consistent SHAPE reactivity pattern across all three genomes: Adenines show high reactivity, and the guanine nucleotides are unreactive (Fig 7A). The RNA regions spanning the cPPT and PPT also showed strong statistical interdependencies in whole genome alignments (Fig 2). Both the cPPT and PPT, contain conserved base pairs in consensus structural models (Fig 7B), and the cPPT and PPT regions show striking structural similarities in their predicted models. The 3ʹ end of the G-rich region forms a structurally conserved helix. The helix forms the boundary for a single-stranded region containing the A-rich 5ʹ end of the polypurine sequence. This single-stranded region contains short helical elements that are conserved in consensus models for the PPT (Fig 7B).

This conserved structure may contribute to the known function of the PPT tract as an RNA primer for second-strand DNA synthesis. Consensus cPPT/PPT structures are conspicuously positioned near the RNase H cleavage site, suggesting a possible connection between RNA structure and recognition of the PPT by the RNase H domain of reverse transcriptase. The 3ʹ end of the PPT is within a helical element. RNA secondary structure has been shown to influence polymerization-dependent RNA cleavage by inducing pausing of the reverse transcriptase [42, 43]. Although this element would not account for cleavage precisely at the 3ʹ end of the PPT [41], secondary structure-dependent cleavage during reverse transcription may be a first step in the processing of the PPT RNA primer. These conserved RNA structures may also have additional functions.

### Perspective

With advances in high-throughput chemical probing, interrogation of RNA structure at the transcriptome level is now possible. Structured motif discovery and annotation requires rigorous and accurate approaches for automated RNA structure characterization and motif discovery. Using structure-based alignments derived from SHAPE reactivities, we identified statistically correlated RNA structure motifs conserved across related viral genomes and then modeled secondary structures for these regions based on both sequence covariation and chemical probing-derived structural information. The resulting structures recapitulated all known functional elements in the HIV-1 RNA genome. Consensus base pairs were also discovered in structural elements with no currently known function; these are outstanding targets for future functional analysis. The ideas and general approaches described here provide a framework for functional RNA structure discovery at the RNA genome and transcriptome levels.

### Availability of SHAPE probing data, alignments, and structure models

All SHAPE-MaP data, alignments, and secondary structure models developed in this work are fully available both in the Supporting Information and at the corresponding author's web page http://www.chem.unc.edu/rna/.

## Methods

### Virus production and genomic RNA purification

Viruses were produced and genomic RNA purified as described [21]. Virus inocula were generated by transfection of the following plasmids into 293T cells (using TransIT 293, Mirus Bio). HIV-1 was derived from pNL4-3 (GenBank accession no. AF324493; obtained through the NIH AIDS Reagent Program, Division of AIDS, NIAID, NIH, from Dr. Malcolm A. Martin) [14]. The proviral plasmid containing SIVcpz MB897 (GenBank accession no. JN835461) was a gift from Brandon F. Keele, AIDS and Cancer Virus Program [15]. The plasmid containing the SIVmac239 provirus (GenBank accession no. M33262) was obtained through the NIH AIDS Reagent Program, Division of AIDS, NIAID, NIH, from Dr. Ronald C. Desrosiers) [16, 17]. During genomic RNA extraction, care was taken to avoid denaturation of RNA structure by heat or treatment with chaotropic agents. Following lysis with SDS and proteinase K, viral RNA was extracted three times using 25:24:1 phenol/chloroform/isoamyl alcohol, followed by two extractions with pure chloroform. Viral RNA was precipitated in 70% (vol/vol) ethanol with 300 mM KCl and stored at -80°C until use.

### Characterization of genomic RNAs by SHAPE-MaP

Tubes containing roughly 10 μg of precipitated SIVcpz or SIVmac RNA in 70% ethanol were spun in a microfuge at 4°C for 45 min to pellet RNA. Ethanol was removed, and the pellets incubated at room temperature for 10 min to allow remaining ethanol to evaporate. The pellets were resuspended in 20 μL genome resuspension buffer [50 mM HEPES (pH 8.0), 200 mM potassium acetate], and the resulting solution was characterized by absorption spectroscopy at 260 nm to determine RNA concentration.

To determine SHAPE reactivities, three experiments were performed with SIVcpz and SIVmac samples: 1M7 modification of natively-folded RNA, a no-modification background control, and 1M7 modification of denatured RNA [9]. For 1M7 modification of natively-folded RNA and no-modification background controls, aliquots containing 1 μg of SIVcpz or SIVmac RNA were taken from precipitated RNA stocks. To these 1-μg aliquots, 3 μL of 100 mM MgCl_2_ were added, and the RNA solution was brought up to a volume of 90 μL using genome resuspension buffer. The RNA solution was then incubated at 37°C for 15 min before adding 10 μL of 100 mM 1M7 in DMSO (1M7 modification of natively folded RNA) or 10 μL neat DMSO (background control). The RNA solution was then incubated at 37°C for 3 min to allow complete reaction of 1M7. The RNA solution was then held on ice until purification.

For 1M7 modification of denatured RNA, an aliquot containing 1 μg of SIVcpz or SIVmac RNA was taken from precipitated RNA stocks. To this aliquot, 25 μL of 4 denatured control buffer was added [200 mM HEPES (pH 8.0), 16 mM EDTA], and the RNA solution was brought to a volume of 40 μL using nuclease-free water. To this RNA solution, 50 μL of deionized formamide was added. The RNA solution was held at 95°C for 1 min and then added to 10 μL 100 mM 1M7 in DMSO. The reaction was held at 95°C for 1 min before transferring the reaction to ice. The RNA solution was held on ice until purification.

RNA was then purified by affinity binding (RNeasy Min-Elute kit; Qiagen). Following purification, sequencing libraries were generated as described [9], and sequencing output was analyzed using the SHAPE-MaP pipeline [9]. Background mutation rates were high for the first 200 nucleotides of the SIVmac genome, resulting in unusual negative peaks for this region; SHAPE values for the first 200 nucleotides of SIVmac239 were therefore taken from prior work [11].

### SHAPE-structure dependent alignments of genomic RNA

SHAPE-structure dependent alignments were performed as described [23]. Pairwise sequence alignments were generated by dynamic programming, using a SHAPE value comparison scoring metric. Pairwise sequence alignments were then used to create a multiple sequence alignment using T-Coffee [24] by considering only the pairwise SHAPE-dependent alignments. This approach does not use sequence identity and, instead, relies on matched sequence positions in the input alignments.

### Multi-variable linear regression and statistical analysis

SHAPE data were fit using least squares, where *Y* represents HIV-1 SHAPE values and *X*_*1*_ and *X*_*2*_ represent SIVcpz and SIVmac SHAPE values, respectively:

$$Y=\beta_{0}+\beta_{1}X_{1}+\beta_{2}X_{2}+\varepsilon_{i}$$

Correlations between the three HIV-related strains were evaluated using the F-test, which evaluates the interdependency of data sets based upon a linear regression model. The F-test evaluates the following null hypothesis by the sum of squares due to lack-of-fit for the model:

$$\beta_{1}=\beta_{2}=0$$

Based on the derived F-statistic measurements, *p*-values defining the significance of the interdependence of SHAPE values were determined over the entire alignment. In addition, t-tests were used to evaluate pairwise correlations between HIV-1 and either SIVcpz or SIVmac. Statistical analyses were performed over 200-nt windows in the multiple sequence alignment. Over each window, only positions with SHAPE values for each genome were considered; no gapped positions were included. Multi-variable linear regression analyses were performed using the NumPy, SciPy, and statsmodels Python modules [44, 45]. Multi-variable linear models were created using least squares fitting. F-tests and t-tests were performed over each window using statsmodels [45]. We note that there are regions in the whole-genome alignment where the F-test is not significant but where one of the pairwise t-tests is significant. This disagreement likely reflects, in part, correlation between the SIVcpz and SIVmac data sets (collinearity).

### Generation of reference alignment and evaluation of SHAPE-dependent alignment

A manually edited alignment considering diverse primate lentiviruses was taken from the 2012 HIV Compendium (section “Alignment of Primate Lentivirus Complete Genomes”) [27]. A reference multiple sequence alignment containing the NL4-3 sequence was generated by inserting this sequence into the HIV Compendium alignment using MAFFT [46]. Agreement between SHAPE-dependent and reference alignments was evaluated using sum of pairs and column score analyses [47]. Sum of pairs is the percentage of reference matched position pairs recapitulated in an alignment, and column score is the percentage of vertical columns that are shared with a reference alignment.

### Selections of areas of interest for secondary structure prediction

Areas of interest for secondary structure modeling were selected based on F-test statistics of multi-variable linear regression models. If a given 200-nt window had an F-test p-value less than 0.01, the corresponding 200-nt region was selected as an area of interest. Consecutive areas of interest were combined in the same secondary structure element.

### Consensus secondary structure prediction

Secondary structures for the reference HIV-1 sequence were generated with a two-step procedure using RNAalifold and RNAfold, both of the Vienna-RNA software package [29, 32, 48]. First, consensus based pairs were generated using RNAalifold based on the SHAPE-directed sequence alignment. Two consensus secondary structures were predicted for each F-test-defined element. The first consensus considered HIV-1, SIVcpz, and SIVmac sequences. The second consensus considered (the more closely related) HIV-1 and SIVcpz sequences only. Consensus structures were generated using the ribosum substitution matrix and a max base pairing distance of 600 nucleotides [49]. Consensus structure prediction incorporated SHAPE reactivities using a pseudo-free energy change potential [31].

Following consensus model generation, consensus base pairs were used to constrain a single-genome secondary structure prediction for HIV-1. Base pairs from each consensus structure with pairing probabilities greater than 95% were added to a constraint list. The constraint list was curated such that consensus pairs were excluded if either (*i*) pairs with shared nucleotides contradicted each other in terms of base pairing partners or (*ii*) pairs from two consensuses were non-nested. HIV-1 structure predictions constrained by consensus pairs were performed with RNAfold. Predictions were constrained such that curated consensus pairs were maintained in the final structure. SHAPE reactivities were incorporated into secondary structure model using a pseudo-free energy potential [31]; the maximum allowed base pairing distance was 600 nucleotides.

## Supporting Information

S1 Fig Evaluation of agreement between SHAPE-only alignment with a manually curated reference alignment from the LANL HIV compendium [27]. The column score, the percentage of positions (columns) recapitulated in a given alignment, for the SHAPE-dependent alignment relative to the LANL alignment is shown across the entire HIV-1 genome over 200-nt windows. HIV-1/SIVcpz and HIV-2/SIVmac reading frames and the positions of *env* variable loop regions are shown as a function of NL4-3 sequence. Low levels of agreement, reflected by low column score values, occur at regions encoding multiple protein reading frames and regions encoding variable loops in *env*.
(EPS)

S1 Dataset All SHAPE-MaP reactivity data, alignments, and secondary structure models are provided in a single dataset archive. (ZIP)
